# Implementing Brief Tobacco Cessation Interventions in Community Pharmacies: An Application of Rogers’ Diffusion of Innovations Theory

**DOI:** 10.3390/pharmacy10030056

**Published:** 2022-05-30

**Authors:** Katy Ellis Hilts, Robin L. Corelli, Alexander V. Prokhorov, Susan M. Zbikowski, Alan J. Zillich, Karen Suchanek Hudmon

**Affiliations:** 1 Richard M. Fairbanks School of Public Health, Indiana University, Indianapolis, IN 46202, USA; kaaellis@iupui.edu; 2 Department of Clinical Pharmacy, School of Pharmacy, University of California San Francisco, San Francisco, CA 94117, USA; robin.corelli@ucsf.edu; 3 MD Anderson Cancer Center, The University of Texas, Houston, TX 77030, USA; aprokhor@mdanderson.org; 4 inZights Consulting, LLC, Seattle, WA 98115, USA; suezbikowski@inzights-consulting.com; 5 Department of Pharmacy Practice, College of Pharmacy, Purdue University, West Lafayette, IN 47907, USA; azillich@purdue.edu

**Keywords:** tobacco, tobacco cessation, smoking, smoking cessation, pharmacist, community pharmacy, quitline, brief intervention

## Abstract

Pharmacists, as highly accessible members of the healthcare team, have considerable potential to address tobacco use among patients. However, while published data suggest that pharmacists are effective in helping patients quit, barriers exist to routine implementation of cessation services in community pharmacy settings. Within the context of a randomized trial (n = 64 pharmacies), surveys were administered over a period of 6 months to assess pharmacists’ perceptions of factors associated with the implementation of “Ask-Advise-Refer”, a brief intervention approach that facilitates patient referrals to the tobacco quitline. Study measures, grounded in Rogers’ Diffusion of Innovations Theory, assessed pharmacists’ perceptions of implementation facilitators and barriers, perceptions of intervention materials provided, and perceived efforts and personal success in implementing Ask-Advise-Refer at 6-months follow-up. Findings indicate that while the brief intervention approach was not difficult to understand or implement, integration into normal workflows presents greater challenges and is associated with overall confidence and implementation success. Lack of time was the most significant barrier to routine implementation. Most (90.6%) believed that community pharmacies should be active in promoting tobacco quitlines. Study results can inform future development of systems-based approaches that lead to broad-scale adoption of brief interventions, including but not limited to tobacco cessation, in pharmacy settings.

## 1. Introduction

### 1.1. Pharmacists’ Role in Tobacco Cessation

In the United States, nearly 70% of adults who smoke express an interest in quitting, yet only 31.2% report using evidence-based cessation interventions to do so, and fewer than 1 of every 10 who attempt to quit are successful [1]. Underutilization of cessation interventions is, in part, due to limited access to health professionals when patients are ready to quit [2]. Patient barriers include delays in the healthcare system to obtain an appointment with a family medicine physician (e.g., an average of 29.3 days [3]), transportation issues, and inability to schedule appointments outside of standard work hours [4]. Pharmacists represent an important community-based healthcare provider pool with considerable potential for reducing tobacco use among the general population [5]. However, although pharmacists are interested in helping patients quit using tobacco products [6,7], and published data suggest that they can be both effective [8,9] and cost-effective [10], time constraints in community pharmacy settings impact the feasibility of integrating comprehensive tobacco cessation services into practice [11,12]. As an alternative, it has been hypothesized that brief interventions—asking patients whether they use tobacco products, advising tobacco users to quit, and referring those who are ready to quit in the next month to other cessation resources (i.e., Ask-Advise-Refer)—might be a feasible alternative in busy pharmacy settings [13].

Published research suggests that brief interventions are effective in connecting patients to cessation services in primary care [14] and behavioral health settings [15], and in emergency departments [16]. Furthermore, prior research suggests that brief interventions are a feasible approach to delivering tobacco cessation in the context of community pharmacies [17,18,19]. However, few studies have applied theory to assess factors that support or impede the adoption of brief interventions for tobacco cessation into routine practice in community pharmacy settings [20]. Furthermore, to our knowledge, no studies have explored perceptions of materials designed specifically to support the implementation of pharmacy-based tobacco cessation services.

### 1.2. Brief Interventions for Tobacco Cessation in Community Pharmacies: Ask-Advise-Refer

The “Ask-Advise-Refer” project was designed to facilitate brief tobacco cessation interventions provided through a team approach involving community pharmacists and pharmacy technicians. In this project, Rogers’ Diffusion of Innovations Theory [21] served as a guiding framework for (a) developing the intervention components and implementation materials, (b) operationalizing two strategies for engaging pharmacy staff, and (c) setting the stage for the future study of broad-scale adoption of the concept. Diffusion of Innovations can be described as “the process by which an innovation is communicated through certain channels over time among members of a social system [21].” Stages through which innovations evolve include dissemination, adoption, implementation, and institutionalization. The rate of diffusion of an innovation is dependent upon attributes of the innovation, characteristics of the adopter, the chosen channels of communication for disseminating the innovation, and the social system. The theory posits that the adoption of an innovation (e.g., the Ask-Advise-Refer practice model) is affected by the stakeholders (e.g., pharmacists, technicians, physicians, patients), the unit of decision-making (group, authoritative, collective), the potential differences that exist between program adopters and implementers, and the need for existing organizational structures to be compatible with and able to support the proposed change.

The goal of this study was to estimate the extent to which the “innovation” (i.e., the Ask-Advise-Refer practice model), implemented as part of a randomized trial, is adoptable in community pharmacy settings. According to Rogers [21], specific qualities determine the success of an innovation, such as being compatible with current practices, reliably advantageous, trialable and observable, and of minimal complexity to allow for implementation into routine clinical practice. Thus, the purpose of the current report, which is a part of a series of papers describing the process and outcomes related to the Ask-Advise-Refer project, is to characterize pharmacists’ perceptions of the factors that facilitate and impede the implementation of the model into practice, and the likelihood of using select implementation materials. Lastly, we assessed correlations between pharmacists’ perceptions of the factors that facilitate or impede implementation and their perceived efforts and success in implementing the Ask-Advise-Refer approach into routine practice. Results of this study can inform future broad-scale adoption of brief interventions to support pharmacy-based services, including, but not limited to, tobacco cessation.

## 2. Material and Methods

### 2.1. Study Design

This study applied a randomized design, with the community pharmacy as the unit of randomization, to estimate the impact of two intervention strategies for engaging community pharmacy personnel in referring patients who use tobacco and are interested in quitting to the tobacco quitline [19,22,23]. Quitlines are publicly funded programs available in all 50 states and US territories (1-800-QUIT NOW), offering a variety of free resources, including information on how to quit smoking, 1-on-1 telephone-based counseling, referral to other cessation resources, information on FDA-approved cessation medications, and in some cases, cessation medications provided at no cost [24]. A robust recruitment approach [22] yielded the targeted total of 32 pharmacies in Connecticut (CT) and 32 pharmacies in Washington (WA). Through stratified sampling and recruitment, half of the pharmacies were independently-owned, and half were retail chain pharmacies.

**Figure 1:**
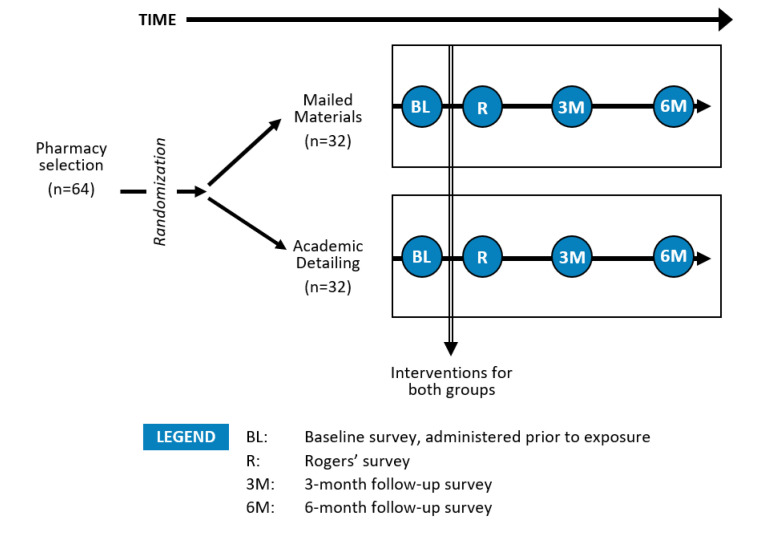
Study design and survey assessments. Adapted with permission from ref. [23] Copyright 2022 Elsevier.

The 64 participating sites were randomized into 1 of 2 intervention arms: (1) academic detailing (AD) or (2) mailed materials (MM) (Figure 1). All pharmacists and technicians employed within the pharmacies were invited to participate, and four written surveys were distributed to participants: the baseline survey (prior to the intervention), the Rogers’ survey (immediately after receiving the quitline materials from the academic detailer or by mail), and at 3- and 6-months follow-up. Visa gift cards were provided as a form of compensation. Participants who completed the baseline and Rogers’ surveys received USD 20; an additional USD 20 was provided for the 3-month survey, and USD 40 for the 6-month survey. The “primary pharmacist contact” at each store received an additional USD 10 for completing a separate survey assessing the characteristics of the pharmacy setting. 

Because the study was designed to maximize effectiveness (e.g., emulating the “real world” to the extent possible), the interventions were implemented in a manner that minimized investigator impact on outcomes. Consistent with this, except for addressing reorders for implementation tools, the research team communicated with the study sites only during periods of data collection. Detailed aspects of the study design, including sampling procedures, recruitment results, and baseline findings, are described in detail elsewhere [19,22,23]. The study procedures and materials were approved by the Purdue University Human Research Protection Program.

### 2.2. Intervention Arms

The two intervention arms each represented an approach that was hypothesized, in collaboration with our tobacco quitline partners, to be feasible and “packageable” for broad-scale dissemination of the Ask-Advise-Refer practice model to community pharmacies across the US. Implementation materials that were provided to the pharmacies are delineated in Table 1; these were parallel between groups, with the notable exception that pharmacies in the AD arm received brief, on-site training for implementing Ask-Advise-Refer, along with (a) digital videos demonstrating the use of the Ask-Advise-Refer approach in a community pharmacy and (b) the ability to submit faxed referrals to the tobacco quitline, which then prompted the quitline to contact the patient directly to initiate enrollment in their telephone-based cessation program.

### 2.3. Study Measures

Key measures reported here include (a) pharmacy and pharmacist characteristics, (b) pharmacists’ perceived characteristics of the Ask-Advise-Refer approach, (c) anticipated use of implementation materials that were provided by the study team, and (d) barriers to implementing Ask-Advise-Refer in their pharmacy setting, and (e) self-reported personal efforts and success in implementing Ask-Advise-Refer at 6-months follow-up. Except where otherwise indicated, all measures were derived from the Rogers’ survey (i.e., after exposure to the intervention; Figure 1).

Pharmacy and Pharmacist Characteristics: In the baseline survey, pharmacists reported sociodemographic characteristics, practice characteristics (e.g., years worked at current pharmacy and hours worked per week), past training for tobacco cessation, and current practices (e.g., percent of patients asked about tobacco use). In the additional survey that was sent to each pharmacy at baseline, the pharmacy manager or primary contact reported the average number of prescriptions filled, availability of private or semi-private counseling rooms, and data related to tobacco cessation counseling activities (e.g., processes to identify patients who smoke, if patients are routinely asked about smoking, and the percentage of new and existing patients who are asked about tobacco use).

Perceived Characteristics of the Ask-Advise-Refer Approach: Mapping onto key constructs hypothesized to be associated with the adoption of an innovation [21], survey items were created to capture pharmacists’ perceptions of the brief intervention model. These constructs include: (a) compatibility of the Ask-Advise-Refer approach with the daily workflow in the pharmacy, (b) advantage of the approach over other tobacco cessation counseling approaches, (c) acceptability of the approach for implementing into routine practice, (d) appropriateness of the approach for use in your pharmacy, and (e) clarity of the three steps (Ask, Advise, and Refer). Pharmacists rated each of these characteristics using a 4-point scale: 1 = none, 2 = low, 3 = moderate, and 4 = high. Pharmacists were also asked, “Please rate your confidence in your personal ability to routinely implement the Ask-Advise-Refer approach” and “Please rate your confidence in your pharmacy’s overall ability to routinely implement the Ask-Advise-Refer approach”. For both items, response options were 1 = none, 2 = low, 3 = moderate, and 4 = high.

Anticipated Use of Implementation Materials: To ascertain the value of various implementation materials (Table 1) that were provided, pharmacists in both arms were asked, “Please rate the likelihood that you will use each of the following items in your community pharmacy”. Response options were 1 = none, 2 = low, 3 = moderate, and 4 = high. In the AD group, pharmacists were also asked to rate the overall usefulness of the on-site quitline ‘academic detailing’ training.

Barriers to Implementing Ask-Advise-Refer: To assess perceived barriers to implementation, participants were asked, “Please rate the *importance* of each of the following potential barriers to using the ‘Ask-Advise-Refer’ approach as part of *routine workflow* in your community pharmacy”. Barriers, which were assessed in the Rogers’ survey, included: (a) lack of available time, (b) lack of training, (c) discomfort in asking patients about tobacco use, (d) lack of staff’s perceived importance of tobacco cessation counseling as applicable to their job, and (e) lack of confidence in discussing quitting with patients. Response options were 1 = not at all important, 2 = a little important, 3 = moderately important, 4 = very important, and 5 = extremely important. At the 6-month follow-up assessment, pharmacists reported their level of agreement to the statement, “I have insufficient time to counsel patients about quitting smoking” (response options on a 5-point Likert scale ranged from strongly disagree to strongly agree).

Self-Reported Personal Efforts and Success in Implementing Ask-Advise-Refer: Self-reported survey data collected at 6-months post-intervention were used to assess pharmacists’ perceptions of their efforts and success in implementing the Ask-Advise-Refer approach. Specifically, participants were asked, “How would you rate your *personal efforts* in implementing Ask-Advise-Refer for helping patients quit since the beginning of the study?” and “How would you rate your *personal success* in implementing Ask-Advise-Refer for helping patients quit since the beginning of the study?”. Response options for both questions were 1 = poor, 2 = fair, 3 = good, 4 = very good, and 5 = excellent.

Perceived Role of Pharmacy in Tobacco Cessation: Three dichotomous items were asked at the end of the final, 6-month survey: “Do you believe that the pharmacy profession should become more active in helping patients with quitting smoking?”, “Do you believe that more community pharmacies across the US should become active in promoting 1-800-QUIT NOW?”, and “As a result of being in the study, have you become more interested in the topic of tobacco cessation in general?”. Response options were yes or no.

### 2.4. Statistical Analyses

Standard summary statistics and t-tests were used to characterize and compare group responses, respectively, and Spearman’s rank correlation was computed to assess the relationship between pharmacists’ ratings of the key factors (characteristics of Ask-Advise-Refer and barriers) and self-reported ratings of personal efforts and success in integrating Ask-Advise-Refer into routine practice. Statistical significance was set at *p* < 0.05. Data were analyzed using SPSS Version 28.0 (IBM Corp., Armonk, NY, USA).

## 3. Results

### 3.1. Pharmacy and Pharmacist Characteristics

On average, pharmacies were open 10.9 h per weekday, dispensing an average of 215 prescriptions per day. Most (76.2%) had a semi-private counseling area, 14.3% had a private counseling area, and 9.5% had both. At baseline, 27.4% of pharmacies indicated that they offered smoking cessation counseling to patients. No significant differences in store characteristics were observed by the intervention arm.

Across all 64 pharmacies, of the 120 pharmacists who consented to participate in the study and completed the baseline survey, 114 (95.0%) completed the Rogers’ survey, and 108 (90.0%) completed the 6-month survey. With the exception of age, for which the MM group was 51.0 years (standard deviation [SD], 13.5) compared to 44.2 years (SD, 11.3) in the AD group, no statistically significant between-group differences were observed. More than half of all pharmacists in the current study indicated they had no prior formal training for tobacco cessation. On average, pharmacists reported at baseline that their pharmacy personnel asked 4.6% of all patients (SD, 10.6) about their tobacco use status.

### 3.2. Perceived Characteristics of the Ask-Advise-Refer Approach

The clarity of the three steps comprising Ask-Advise-Refer received the highest overall rating among pharmacists in both intervention arms, with a mean of 3.7 (SD, 0.6). On average, pharmacists provided ratings between 3 (moderate) and 4 (high) for the four other characteristics of the Ask-Advise-Refer approach (Table 2). Similarly, pharmacists rated confidence in their personal ability as 3.2 (SD, 0.7) and their pharmacy’s ability as 3.1 (SD, 0.7) to implement the Ask-Advise-Refer approach routinely. No differences were observed between intervention arms.

### 3.3. Anticipated Use of Implementation Materials

Overall, pharmacists rated the likelihood of using quitline cards (mean, 3.5; SD, 0.7), brochures (mean, 3.6; SD, 0.6), and counter-top display racks (mean, 3.6; SD, 0.7) highest among the implementation materials that were provided. No baseline differences were observed between groups for the likelihood of use for any of the implementation materials (Table 3).

### 3.4. Barriers to Implementing Ask-Advise-Refer

The highest-rated barrier overall was lack of available time (mean, 3.7; SD, 1.1), followed by discomfort in asking patients about tobacco use (mean, 2.7; SD, 1.2), and lack of training (mean, 2.6; SD, 1.2). No statistically significant differences were observed, by group, for any of the barriers (Table 4). At the 6-month follow-up, just over one third either strongly agreed (3.7%) or agreed (32.7%) that they have insufficient time to counsel patients about quitting smoking; 19.6% were not sure, 36.4% disagreed, and 7.5% strongly disagreed.

### 3.5. Self-Reported Personal Efforts and Success in Implementing Ask-Advise-Refer

Overall, pharmacists rated their personal efforts in implementing the Ask-Advise-Refer approach as “fair/good” (mean, 2.5; SD, 1.0) and their personal success slightly lower (mean, 2.3; SD, 1.0). No differences were observed between intervention arms.

Spearman rank correlations revealed statistically significant associations between pharmacists’ ratings of their personal efforts and personal success in implementing Ask-Advise-Refer and several of the Rogers constructs: perceived compatibility with daily workflow (*p*-values < 0.01), the relative advantage over other tobacco cessation approaches (*p*-values < 0.01), and acceptability of implementing Ask-Advise-Refer into routine practice (*p*-values < 0.02) (Table 5).

When considering barriers, self-rated personal efforts and personal success in implementing Ask-Advise-Refer were inversely correlated with lack of available time (*p*-values < 0.05) and discomfort (*p*-values < 0.01) in asking patients about tobacco use. Additionally, lack of confidence in counseling patients about quitting was inversely associated with personal success for Ask-Advise-Refer implementation (*p* < 0.05).

### 3.6. Perceived Role of Pharmacy in Tobacco Cessation

Across all participants, 90.6% believed that pharmacies should become more involved in tobacco cessation, and 98.1% believed that community pharmacies should promote the tobacco quitline (no differences by intervention arm). Additionally, 87.9% reported that they became more interested in tobacco cessation as a result of being in the study.

## 4. Discussion

Previously published, primary outcomes from this randomized trial indicate that pharmacy personnel are highly effective in increasing the overall number of patients who call the tobacco quitline for assistance with quitting [19]. However, the results presented here suggest that changes to system- or organizational-level factors would enhance the more robust integration of tobacco cessation services into community pharmacy practice. Specifically, while pharmacists rated the clarity of the Ask-Advise-Refer approach as high, they considered this model to be somewhat less compatible, acceptable, advantageous, and appropriate for use in practice, with time being the most important barrier. These findings indicate that while the intervention itself is not difficult to understand or to use, the feasibility of integration within normal workflows presents a greater challenge and may affect overall confidence. Consistent with our prior findings [19], there does not appear to be a difference between the two intervention arms, suggesting that either intervention model (MM or AD) would yield comparable results.

Additional findings in the current report further suggest that individual-level factors (e.g., confidence in tobacco cessation counseling, discomfort in asking about tobacco use, etc.) do not appear to impede the adoption of brief tobacco cessation interventions. Although these factors were rated as moderately important perceived barriers at baseline, at the end of the study, pharmacists reported fair/good ratings for their personal success and efforts in implementing the Ask-Advise-Refer model. Statistically significant positive relationships were observed between the compatibility of the approach within the daily workflow in the pharmacy, the advantage of the approach compared to other cessation counseling approaches, and acceptability for implementing into routine practice for both perceived effort and perceived success in implementing the Ask-Advise-Refer model. These findings provide additional support for the need to ensure that tobacco cessation interventions are designed in a way that supports ease of integration into normal practices.

Overall, pharmacists provided high ratings for the likelihood of using quitline cards, brochures, and counter-top displays. Interestingly, initial findings from our previous report [19] found that 42% of callers to the quitline indicated having seen or received a quitline card, 38% recalled seeing or receiving a brochure, and 17% reported seeing a counter-top display with quitline materials, suggesting the potential utility of these support materials in raising awareness. While pharmacists’ initial ratings of quitline stickers for pharmacy bags indicated a lower perceived utility of this resource, it was reported as having been seen by 10% of quitline callers who reported having heard about the quitline at a pharmacy [19]. Understanding pharmacists’ perceptions and use of these implementation materials can help to inform future efforts to support the adoption of services in pharmacy settings.

The integration of tobacco cessation services in pharmacy settings represents a unique opportunity to connect patients to evidence-based interventions. Importantly, our randomized trial resulted in highly significant increases in the number of quitline program enrollees who reported having heard about the quitline at a pharmacy [19]. Furthermore, the systematic integration of tobacco cessation education into pharmacy school curricula [25,26,27], a growing number of states with legislation enabling pharmacists to provide tobacco cessation medications without a prescription from a provider [28], and widespread dissemination of continuing education programs [29,30,31,32] all support pharmacy personnel’s enhanced role in delivering tobacco cessation services. However, as our findings suggest, system-level strategies are needed for pharmacists in community settings to function at the top of their license with respect to tobacco cessation [33]. Our own work provides evidence that pharmacy technicians are essential to the process [31,34] and could alleviate some of the burden on pharmacists with respect to implementing Ask-Advise-Refer. Although we applied Rogers’ Diffusion of Innovations Theory to enhance our understanding of facilitators and barriers, additional research is needed to further expand our knowledge of the contextual factors that support and impede more robust adoption of pharmacy-based tobacco cessation services [20,35].

## 5. Conclusions

Although a variety of effective treatment options exist, nearly two thirds of current smokers try to quit without any proven cessation methods. Among those who do utilize proven methods for quitting, most use medications, with fewer using behavioral support and even fewer using a combination of both [1]. Pharmacies represent a well-trained and accessible community entity with the potential to address tobacco use among diverse population subgroups, and the results of this study indicate that, regardless of the intervention arm, pharmacists became more interested in this patient care activity and believe the profession should be more involved tobacco cessation. Despite pharmacists’ perceived interest and capability, and the brief nature of the Ask-Advise-Refer approach, the time needed to implement tobacco cessation services into practice remains a barrier to more robust implementation. Additional research is needed to identify and evaluate systems- and organizational-level strategies to enhance the capacity for delivery of clinical services, including but not limited to tobacco cessation, in time-constrained community pharmacies.

## Figures and Tables

**Table 1:** Implementation materials provided to participating pharmacies by intervention arm. Reprinted with permission from ref. [23] Copyright 2022 Elsevier.

Implementation Materials	Academic Detailing	Mailed Materials
Cover letter from the state quitline, describing the project and its collaborators/endorsers, including state and national pharmacy associations	✓	✓
A 1-page document describing the efficacy of the quitline and the process that occurs when a patient calls the quitline	✓	✓
Quitline cards	✓	✓
Quitline tri-fold brochures	✓	✓
Plastic counter-top display to hold the quitline cards and quitline brochures	✓	✓
“Ready to Quit? I can help” buttons to be worn on the lapel of pharmacy staff	✓	✓
Quitline stickers for placement on prescription bags	✓	✓
Quitline posters to be placed on walls near the pharmacy counter	✓	✓
*Pharmacologic Product Guide* and *Drug Interactions with Tobacco Smoke* resources for use by pharmacists (laminated)	✓	✓
*How to Implement Ask-Advise-Refer* resource, describing the step-by-step process for use by pharmacists and technicians (laminated)	✓	✓
Re-order form for materials	✓	✓
Quitline fax referral forms	✓	
CD-ROM with videos demonstrating *Ask-Advise-Refer* in a pharmacy setting	✓	
Relevant published literature supporting the concept of the tobacco quitline	✓	

**Table 2:** Pharmacists’ (n = 114) ratings ^a^ of characteristics of the “Ask-Advise-Refer” approach and confidence for implementation within the pharmacy, overall and by intervention arm [mean (SD)].

Characteristics and Confidence	Overall	Mailed Materials (n = 59)	Academic Detailing (n = 61)	*p*-Value
**Characteristics of Ask-Advise-Refer** [21]				
Compatibility of the Ask-Advise-Refer approach with your daily workflow in the pharmacy	3.1 (0.6)	3.2 (0.7)	3.0 (0.6)	0.24
Advantage of the Ask-Advise-Refer approach over other tobacco cessation counseling approaches	3.3 (0.7)	3.2 (0.7)	3.3 (0.6)	0.50
Acceptability of the Ask-Advise-Refer approach of implementing into routine practice	3.2 (0.7)	3.3 (0.6)	3.2 (0.7)	0.56
Appropriateness of the Ask-Advise-Refer approach for use in your pharmacy	3.4 (0.6)	3.4 (0.6)	3.4 (0.6)	0.88
Clarity of the three steps (Ask, Advise, Refer)	3.7 (0.6)	3.6 (0.6)	3.7 (0.5)	0.23
**Confidence in implementing Ask-Advise-Refer**				
Personal confidence in ability to routinely implement AAR approach	3.2 (0.7)	3.2 (0.7)	3.1 (0.7)	0.49
Confidence in pharmacy’s ability to routinely implement AAR approach	3.1 (0.7)	3.2 (0.7)	3.0 (0.6)	0.12

^a^ Survey item was worded as: “Please rate each of the following characteristics of the ‘Ask-Advise-Refer’ approach to tobacco cessation counseling in your community pharmacy.” Response options ranged from 1 = none to 4 = high.

**Table 3:** Pharmacists’ (n = 114) ratings ^a^ of likelihood of using implementation materials within the pharmacy, overall and by intervention arm [mean (SD)].

Implementation Materials	Overall	Mailed Materials (n = 59)	Academic Detailing (n = 61)	*p*-Value
Quitline cards	3.5 (0.7)	3.5 (0.7)	3.5 (0.7)	0.79
Quitline brochures	3.6 (0.6)	3.6 (0.5)	3.6 (0.7)	0.75
Counter-top display	3.6 (0.7)	3.5 (0.8)	3.6 (0.7)	0.48
Pharmacologic product guide	3.2 (0.8)	3.3 (0.7)	3.0 (0.8)	0.08
Drug interactions with smoking table	3.2 (0.8)	3.2 (0.7)	3.1 (0.9)	0.91
“Ready to quit? I can help” buttons	2.6 (1.0)	2.7 (1.0)	2.5 (0.9)	0.41
Quitline stickers for prescription bags	3.0 (0.9)	3.1 (0.8)	2.9 (0.9)	0.19
Quitline posters	3.3 (0.9)	3.3 (0.8)	3.3 (0.9)	0.83

^a^ Survey item was worded as: “Please rate the likelihood that you will use each of the following items in your community pharmacy.” Response options ranged from 1 = none to 4 = high.

**Table 4:** Pharmacists’ (n = 114) ratings ^a^ of the importance of potential barriers to implementing Ask-Advise-Refer, overall and by intervention arm [mean (SD)].

Barriers to Implementing Ask-Advise-Refer	Overall	Mailed Materials (n = 59)	Academic Detailing (n = 61)	*p*-Value
Lack of available time	3.7 (1.1)	3.8 (1.1)	3.6 (1.1)	0.43
Lack of training	2.6 (1.1)	2.8 (1.2)	2.4 (1.0)	0.09
Discomfort in asking patients about tobacco use	2.7 (1.2)	2.8 (1.3)	2.7 (1.1)	0.70
Lack of staff’s perceived importance of tobacco cessation counseling as applicable to job	2.4 (1.1)	2.4 (1.2)	2.5 (1.0)	0.66
Lack of confidence in counseling patients about quitting	2.2 (1.0)	2.2 (1.0)	2.1 (0.9)	0.55

^a^ Survey item was worded as: “Please rate the importance of each of the following potential barriers to using the ‘Ask-Advise-Refer’ approach as part of *routine workflow* in your community pharmacy. Note: If you feel that one or more of the barriers listed *does not apply* to your pharmacy, please rate it as ‘not at all important.’” Response options ranged from 1 = not at all important to 5 = extremely important.

**Table 5:** Relationships between pharmacists’ (n = 108) (a) ratings of characteristics of and barriers to implementing the Ask-Advise-Refer approach and (b) self-rated personal efforts and success in integrating the Ask-Advise-Refer into routine practice ^a^ (assessed at the 6-month follow-up).

Rogers’ Characteristics and Barriers	Personal Efforts (*r_s_*)	Personal Success (*r_s_*)
**Characteristics of Ask-Advise-Refer** [21]		
Compatibility of the Ask-Advise-Refer approach with your daily workflow in the pharmacy	0.39 ^b^	0.36 ^b^
Advantage of the Ask-Advise-Refer approach over other tobacco cessation counseling approaches	0.29 ^b^	0.32 ^b^
Acceptability of the Ask-Advise-Refer approach of implementation into routine practice	0.27 ^b^	0.21 ^c^
Appropriateness of the Ask-Advise-Refer approach for use in your pharmacy	0.16	0.12
Clarity of the three steps (Ask, Advise, Refer)	0.14	0.12
**Barriers to implementing Ask-Advise-Refer**		
Lack of available time	−0.24 ^c^	−0.21 ^c^
Lack of training	−0.16	−0.18
Discomfort in asking patients about tobacco use	−0.31 ^b^	−0.28 ^b^
Lack of staff’s perceived importance of tobacco cessation counseling as applicable to job	−0.18	−0.17
Lack of confidence in counseling patients about quitting	−0.19	−0.24 ^c^

^a^ Survey items worded as: “How would you rate your *personal efforts* in implementing Ask-Advise-Refer for helping patients quit since the beginning of the study?” and “How would you rate your *personal success* in implementing Ask-Advise-Refer for helping patients quit since the beginning of the study?” Response options for both questions were 1 = poor, 2 = fair, 3 = good, 4 = very good, and 5 = excellent. ^b^ Spearman’s rank correlation is significant at *p* < 0.01. ^c^ Spearman’s rank correlation is significant at *p* < 0.05.

## Data Availability

Not applicable.
